# ASSOCIATION OF COGNITION AND SMARTPHONE SURVEY ATTRIBUTES IN THE ELECTRONIC FRAMINGHAM HEART STUDY

**DOI:** 10.1093/geroni/igac059.2958

**Published:** 2022-12-20

**Authors:** Chathurangi Pathiravasan, Alexa Beiser, Sudha Seshadri, Claudia Satizabal, Yuankai Zhang, Xuzhi Wang, Chunyu Liu, Joanne Murabito

**Affiliations:** Boston University School of Public Health, Boston, Massachusetts, United States; Boston University School of Public Health, Boston, Massachusetts, United States; University of Texas Health Sciences Center, San Antonio, Texas, United States; Glenn Biggs Institute for Alzheimer’s & Neurodegenerative Diseases, University of Texas Health Sciences Center, San Antonio, Texas, United States; Boston University School of Public Health, Boston, Massachusetts, United States; Department of Biostatistics, Boston University School of Public Health, Boston, Massachusetts, United States; Boston University School of Public Health, Boston, Massachusetts, United States; Boston University, Boston, Massachusetts, United States

## Abstract

Mobile technology offers a remote method to monitor health in older adults and it may provide a platform for early detection of cognitive decline. We aimed to examine attributes of smartphone survey use in the electronic Framingham Heart Study (eFHS) cohort in relation to cognitive testing performed at the time of enrollment. eFHS participants who returned smartphone surveys and underwent cognitive testing were considered in the study (n=1810). The CERAD recall score, Victoria Stroop test interference score, and dichotomous AD8 and MOCA (MOCA score ≤ 19, AD8 score ≥2) were considered as primary exposures. App-based survey adherence was defined as a dichotomous outcome based on whether at least one survey was completed at each 3-month period from baseline to 12 months. Several time attributes were considered including survey return time, touch time, step time, and question completion time. Linear mixed models (LMM for time attributes outcomes and generalized LMM for adherence outcome) were fitted for each cognitive score as the predictor adjusting for age, sex, race/ethnicity, and education level. Results suggest that higher CERAD recall scores were associated with higher odds of completing surveys. There was a significant association between all cognitive exposures and survey time attributes. Participants with poorer cognitive function (lower CERAD, higher stroop interference, MOCA score ≤ 19, AD8 score ≥2) had delayed survey return times, higher touch time, higher step time and higher question completion time. This study contributes to the growing body of evidence that smartphones may be an important tool to identify cognitive decline.

